# Detecting and Preventing Fraudulent Participation in Qualitative Research: Content Analysis of Two Multisite Studies

**DOI:** 10.2196/87037

**Published:** 2026-07-03

**Authors:** Destiny Harden, Nicolette Rodriguez, Kristi Roybal, Tara Coffin, Gina Johnson, Kelley Le Beaux, Maria Connolly, Jennifer Rountree, Chinedu Ukaegbu, Anna C Revette, Brett Nava-Coulter, Alyson Caruso, Jane Roberts, Suzanne Brodney, Kimberly Schoolcraft, Sapna Syngal, David A Drew, Folasade P May, Jennifer S Haas, Staci J Wendt, Erica T Warner

**Affiliations:** 1 Clinical Translational and Epidemiology Unit, Mongan Insitute, Massachusetts General Hospital, 100 Cambridge Street, Boston, MA, 02114, United States, 1 617 724 9516; 2 Division of Gastroenterology, Brigham and Women's Hospital, Boston, MA, United States; 3 Division of Cancer Genetics and Prevention, Dana-Farber Cancer Institute, Boston, MA, United States; 4 Division of Population Sciences, Dana-Farber Cancer Institute, Boston, MA, United States; 5 Health Research Accelerator, Providence Research Network, Renton, WA, United States; 6 WIRB-Copernicus Group Institutional Review Board, Cary, NC, United States; 7 Health Promotion and Disease Department, Great Plains Tribal Health, Rapid City, SD, United States; 8 Survey and Qualitative Methods Core, Dana-Farber Cancer Institute, Boston, MA, United States; 9 Division of General Internal Medicine, Massachusetts General Hospital, Boston, MA, United States; 10 Research Advocacy Training and Support Program, Fight Colorectal Cancer, Springfield, MO, United States; 11 Department of Medicine, Division of Gastroenterology, Massachusetts General Hospital and Harvard Medical School, Boston, MA, United States; 12 Vatche and Tamar Manoukian Division of Digestive Diseases and Jonsson Comprehensive Cancer Center, David Geffen School of Medicine, University of California, Los Angeles, Los Angeles, CA, United States; 13 Department of Medicine, Division of Gastroenterology, VA Greater Los Angeles Healthcare System, Los Angeles, CA, United States

**Keywords:** fraudulent participants, imposters, bots, qualitative research, online recruitment, data accuracy, fraud prevention, social media, digital health, internet

## Abstract

**Background:**

The use of web-based approaches to identify, recruit, enroll, survey, and interview health-related research participants has increased over time, with rapid acceleration since the COVID-19 pandemic. These approaches can make research more accessible to a broader population, but also increase the risk of fraudulent or imposter participants infiltrating research studies. While this threat has been discussed extensively in quantitative survey research, less has been reported in qualitative and mixed methods studies.

**Objective:**

This study aims to identify recurring patterns of fraudulent study participation and to offer strategies for identification, remediation, and reporting.

**Methods:**

Encounters with fraudulent or imposter individuals during recruitment, enrollment, survey distribution, data collection, and focus group sessions in 2 multisite qualitative and mixed methods research studies are presented. Content from both studies was analyzed to identify common themes and develop strategies for prevention and remediation.

**Results:**

Investigators across 2 multisite studies observed several indicators of suspected fraudulent activity, including large response volumes over a short period, highly repetitive email addresses, higher-than-expected proportions of phone numbers with area codes outside the study area, and unusual email/phone responses using atypical language and phrasing. Several imposter or fraudulent individuals disrupted online focus group sessions. To mitigate these issues, both studies implemented remediation strategies, including enhanced screening procedures at baseline, cross-checking of survey responses, and additional identity verification methods prior to participation. Studies took various actions to address these experiences, including notifying the institutional review board, recruitment platforms, and funders.

**Conclusions:**

This multisite study identified multiple ways that imposter or fraudulent participants can pose a significant and evolving threat to the integrity of qualitative and mixed methods. These types of fraudulent actors can distort data and undermine research credibility. Lessons learned highlight the importance of real-time recruitment and enrollment analysis and the need for transparent reporting. Addressing this issue will require a comprehensive approach to prevent and address fraudulent study participation that includes collaboration with multiple stakeholders and the broader research community to effectively address this issue.

## Introduction

Traditional methods of in-person research recruitment, enrollment, and patient interaction created barriers to broad research participation because they required travel and imposed time constraints for researchers and participants alike [1]. These barriers ultimately resulted in reliance on convenience samples in many studies. To counter this, the use of web-based approaches to identify, recruit, enroll, survey, and interview research participants has grown over time and was significantly accelerated during the COVID-19 pandemic [2]. Now, remote recruitment, consent, enrollment, and data collection [3] through digital tools, such as web-based surveys and virtual focus groups, use of social media for recruitment, and use of online resources such as Research Match (Vanderbilt University Medical Center) and Amazon MTurk to identify study participants are common [4-9]. Monetary incentives are also widely used to encourage participation and remunerate participants for their time [10,11]. These approaches have made research more accessible across diverse geographies and participant backgrounds and can broaden reach and expand inclusivity while decreasing the overall cost of research recruitment and participation [5]. Yet they are not without their downsides. First, while these strategies may broaden access for some, the digital divide means others may experience new, previously unappreciated, barriers to access [4]. Second, and most importantly, these approaches may leave studies vulnerable to threats unique to virtual research, including duplicate enrollment, imposters, and fraudulent research participation [4].

Remote study methods mean less face-to-face contact and fewer opportunities to verify participant identity and eligibility. This creates opportunities for infiltration from bots, software programs that perform automated tasks such as completing surveys and eligibility screeners, or imposters and individuals that may join virtual focus groups without meeting eligibility in an attempt to fraudulently participate in studies for monetary gain [12]. Either may skew data, undermine the validity of research findings, and challenge the credibility of results, ultimately eroding trust in the broader research system [4,7]. For example, in a cross-sectional descriptive study that recruited participants through Facebook (Meta Platforms, Inc) and X (formerly known as Twitter, X Corp), researchers found that 94.5% of eligibility screener responses captured through a Research Electronic Data Capture (REDCap; Vanderbilt University) [13,14] survey were fraudulent, with many participants using virtual private networks to mask their location and misrepresent their eligibility [15]. In another study, Medero et al [12] examined 2 qualitative case studies where imposter participants were identified during recruitment and data collection, using those incidents to underscore the importance of implementing practical safeguards and advocating for institutional guidance to preserve research integrity. Such cases highlight the pressing need for new approaches to prevent fraud and ensure the generation of valid scientific products.

Prior publications in this area have primarily focused on web-based surveys and large datasets. However, there has been a growing number of qualitative and mixed methods studies reporting the presence of fraudulent (or imposter) participants within their studies [6,12,16-19]. The presence of fraudulent data in qualitative research is particularly problematic because these studies often rely on smaller, more purposively selected samples [7,15]. Even just a few fraudulent participants can distort the interpretation of themes and insights, leading to incorrect conclusions about the studied phenomenon [7,12]. This paper will discuss the infiltration of bots and imposters in qualitative and mixed methods research across multiple studies and sites, offering a comprehensive analysis that provides lessons learned and offers strategies for prevention and identification of fraudulent participants and data in future studies.

## Methods

### Study 1: Improving Participation of American Indian, Black, and Hispanic/Latino Individuals in Biospecimen Research

#### Study Design

Study 1 was a qualitative focus group study nested within a larger pragmatic trial of stool-based colorectal cancer screening approaches in community health centers and community settings in Boston, Los Angeles, and South Dakota [20,21]. Focus groups were designed to examine knowledge, attitudes, and beliefs regarding biospecimen research among American Indian, Black, and Hispanic/Latino/a individuals in the 3 locations. Participants were eligible if they were between the ages of 45 and 74 years, English or Spanish speakers, self-identified as Black/African American, Latino/a and/or American Indian, and were due for colorectal cancer screening. Recruitment in South Dakota was specifically focused on American Indian participants. Participants were recruited between January 2023 and May 2024 [20]. A total of 21 focus groups were conducted across all sites with 101 participants enrolled.

#### Recruitment

In Boston and Los Angeles, potentially eligible individuals were invited to participate through electronic messages sent through the health system’s patient portal. In Boston, potentially eligible individuals were identified and invited to enroll in the study on Research Match, a national health volunteer registry that was created by several academic institutions and supported by the US National Institutes of Health as part of the Clinical Translational Science Award program [8]. The study was also advertised on an institutional research recruitment platform, which served as a centralized system for enrolling individuals in clinical and nonclinical research studies (the instrument can be found in Multimedia Appendix 1).

In Los Angeles, in addition to the strategies above, the team worked with community leaders who were convened as part of efforts to promote colorectal cancer screening in neighborhoods to identify physical and virtual spaces for recruitment. Based on recommendations from those discussions, paper flyers (the instrument can be found in Multimedia Appendix 1) were distributed in community settings (eg, barber shops and churches) and at community events (eg, health fairs or farmer’s markets). Community leaders also received an electronic copy of the flyer to distribute. Each electronic or printed flyer included information about the study, a QR code, and a link directing interested individuals to a study landing page on REDCap. Interested individuals were invited to complete a REDCap survey to confirm their eligibility, provide their contact information, indicate when and how (ie, phone or email) a research staff member should follow up with them, and provide preferences for focus groups (ie, virtual or in-person setting [Los Angeles only]).

In South Dakota, recruitment efforts targeted 2 communities, Rapid City and Rosebud. Health center staff and investigators collaborated with community leaders to promote and distribute study-related materials through trusted channels. Recruitment strategies in Rapid City (Oyate Health Center) involved the distribution of printed flyers (the instrument can be found in Multimedia Appendix 1) at community centers and frequently visited locations, while in Rosebud, social media outreach via Facebook was used to reach individuals in more remote areas with limited communication options. Potential participants in South Dakota contacted the research staff via telephone, where they were screened for eligibility using the REDCap survey, with responses manually entered in the database by the staff.

#### Data Collection

For all sites, verbal consent to enroll was obtained via phone or in person and documented. Participants were then scheduled for a 60‐90-minute virtual focus group conducted via Zoom (all sites; Zoom Communications, Inc), Microsoft Teams (all sites; Microsoft Corp), or in person (Los Angeles only). Participants were offered a US $50 gift card after completing a focus group.

### Study 2: Increasing Engagement in Genetic Testing for Cancer

#### Study Design

Study 2 used mixed methods to capture patient opinions about engaging in genetic testing, cancer prevention, and early detection, including via remote genetic education and testing services at a single study site [22]. Its research goal was to use information gleaned from the surveys and cognitive interviews to optimize delivery of a remote health care delivery service in underserved populations. Participants were eligible if they were 18 years or older, lived in the United States, self-identified as Black and/or Latino/a race or ethnicity, and had a personal/family history of cancer or were a community leader who reported serving predominately Black and/or Latino/a communities.

#### Recruitment

To recruit, the study team collaborated with pancreatic research programs at other cancer centers and patient advocacy groups. These organizations shared information regarding the study and a link to the eligibility questionnaire via online social media platforms, including Facebook and X, and through e-newsletters (the instrument can be found in Multimedia Appendix 1). Potential participants then completed an online REDCap eligibility questionnaire and provided an email address and phone number for contact purposes, indicating their preferred contact method. After eligibility confirmation, participants were contacted using their preferred mode of communication to complete study questionnaires on REDCap. Once study questionnaires were completed, a member (NR) of the study team contacted the participant to schedule a 1:1 cognitive interview or focus group via Zoom.

#### Data Collection

All 1:1 cognitive interviews and focus groups were 45‐60 minutes in length and conducted by 2 study team members (NR and TC), including a trained qualitative methodologist. After completion of all surveys and the cognitive interviews, participants were remunerated with a US $50 gift card. A total of 55 surveys were completed, and only 24 of those participants completed a focus group or 1:1 interview session.

### Ethical Considerations

Study 1, “Improving participation of American Indian, Black, and Hispanic/Latino Individuals in Biospecimen Research,” obtained institutional review board (IRB) approval in Boston (Mass General Brigham IRB #2022P002546), Los Angeles (Providence IRB #STUDY2022000553), and in South Dakota by the Great Plains IRB #23-R-14GP and by the Sicangu Oyate Research Review Board (SORRB; no protocol number). Individual participant data will not be shared. All participants provided written or verbal consent and were remunerated with a US $50 gift card.

Study 2, “Increasing Engagement in Genetic Testing for Cancer: Dana-Farber/Harvard Cancer Center,” obtained Dana-Farber Cancer Institute IRB approval in Boston (Dana-Farber Cancer Institute IRB #21‐209). Individual participant data will not be shared. Informed consent was obtained from all individuals who participated in this study. Participants were remunerated with a US $50 gift card.

## Results

### Bots, Imposters, and Fraudulent Participants: Experiences at Sites

Several early observations led investigators to suspect potential fraudulent study participation across the study sites. Study 1’s Boston site noticed a high prevalence of Google Voice (Google LLC) numbers provided by interested individuals responding to ads posted on the internal institutional research website as their preferred contact method (Table 1). Additionally, notable similarities in language and tone were observed during phone conversations with participants, particularly when speaking with individuals who had been referred by another participant. The Los Angeles site noticed an influx of responses via their social media recruitment platforms and a preponderance of potential participant phone numbers with area codes outside the intended geographic recruitment area. Both Boston and Los Angeles researchers noticed use of email addresses following a highly repetitive pattern (eg, name+number@gmail.com; Table 2).

**Table 1.** Study-level experiences with potential imposters participants identified during 2 multisite studies among American Indian, Black, and Hispanic/Latino participants in Boston, MA; Los Angeles, CA; and South Dakota, 2022-2024.

Location	Fraud prevention tools enabled at baseline	Indicators of potential fraud	Strategies implemented during the study	Impact
Study 1: improving participation of American Indian, Black, and Hispanic/Latino individuals in biospecimen research				
Boston, MA	CAPTCHA^a^ tool enabled in REDCap^b^ Required enrollment call with study coordinator and an email/mailing address to send participant materials Used trusted internal/external recruitment platforms for recruitment	Rapid completion of REDCap eligibility screener Similarities between phone conversations with “different” participants Influx of interested individuals with Google Voice (Google LLC) numbers and similarly formatted (eg, name+number@gmail.com) email addresses Duplicate submissions across recruitment platforms Influx of interested individuals with Google Voice numbers	Monitored survey completion times and survey submissions that occurred during the same period Reviewed phone numbers and email addresses for all potentially eligible individuals Requested alternative contact info for anyone using Google Voice or with suspicious email addresses Reported experiences to institutional recruitment platform	Fewer participants contacted for follow-up Did not return phone calls to suspected fraudulent individuals Did not return phone calls to suspected fraudulent individuals Participants were excluded and labeled as spam on those platforms
Los Angeles, CA	CAPTCHA tool enabled in REDCap Required participants to turn on cameras during focus group sessions	Unexpectedly large and rapid response to web-based eligibility screener Refusal to turn on camera during focus group or camera on but only a black screen displayed. Refusal to speak during focus group and responses sent via chat used web-based or artificial intelligence–generated content	Stopped posting recruitment links on social media Required all participants to be camera on and fully participate during the focus group session to receive the gift card	Fewer submissions received via REDCap survey Eligible participants had to complete an additional verification call with a study team and agree to be on camera for the focus group session
South Dakota	Required enrollment and eligibility questionnaire to be completed via a phone call with study coordinator	Received responses via phone call and email that were not consistent with the dialect and language of the Native American community	Asked potentially fraudulent users to provide study team with more information	Did not return phone calls or respond to emails from suspected fraudulent individuals
Study 2: increasing engagement in genetic testing for cancer				
Boston, MA	CAPTCHA tool enabled in REDCap	Influx of interested individuals with similarly formatted (eg, name+number@gmail.com) email addresses Increase in survey completions in a short period	Added information to protocol about participant verification Reported experiences to IRB^c^	Two-step verification to match survey responses with answers in focus group sessions IRB supported excluding potentially fraudulent data from analysis, but all participants received remuneration

a CAPTCHA: completely automated public turing test to tell computers and humans apart.

b REDCap: Research Electronic Data Capture.

c IRB: institutional review board.

**Table 2.** Types of fraudulent participants and indicators.

	Description	Indicators
Duplicate enrollee	Eligible person attempts to enroll in study multiple times. Note that not all duplicate enrollments are due to fraud	May enroll across multiple research platforms or complete the eligibility screener multiple times using the same identifiers May enter waiting area or virtual meeting room with camera off and no recognizable name
Ineligible or unverified enrollee	Person that does not meet study eligibility criteria attempts to enroll. May provide inaccurate responses to be deemed eligible. Uninvited participants that may have received a link to access a focus group from a legitimate participant	May use email addresses of a format like this: name+number@gmail.com May use a phone number that is outside of the study area code or use a phone number service like Google Voice May enter waiting area or virtual meeting room with camera off and no recognizable name
Bot	Computer program or network of computers completes web-based screeners or surveys.; may also attempt to join virtual focus groups.	May use email addresses of a format like this: name+number@gmail.com May use a phone number that is outside of the study area code or use a phone number service like Google Voice May enter waiting area or virtual meeting room with camera off and no recognizable name
Meeting crashers	An unauthorized and disruptive intrusion into an online meeting by individuals	Will join a meeting and have no prior knowledge of what it is about May enter waiting area or virtual meeting room with camera off and no recognizable name

During virtual focus groups, there were additional irregularities. Study 1’s Los Angeles site had several participants that refused to turn on their cameras during the session. When prompted to do so, they either exited the meeting entirely or turned their cameras on, only to display a blank screen. We also noted that responses to focus group discussion questions (eg, “What do you know about colorectal cancer?”) provided by participants appeared to be copied verbatim from online sources (eg, “Colorectal cancer is a cancer that starts in the colon or rectum”), which was confirmed through internet searches revealing exact matches to publicly available content. Further suspicion arose when responses exhibited patterns consistent with artificial intelligence–generated text. These patterns, when analyzed in real-time by the researchers, resulted in the focus group session ending earlier than expected.

**Figure 1.**
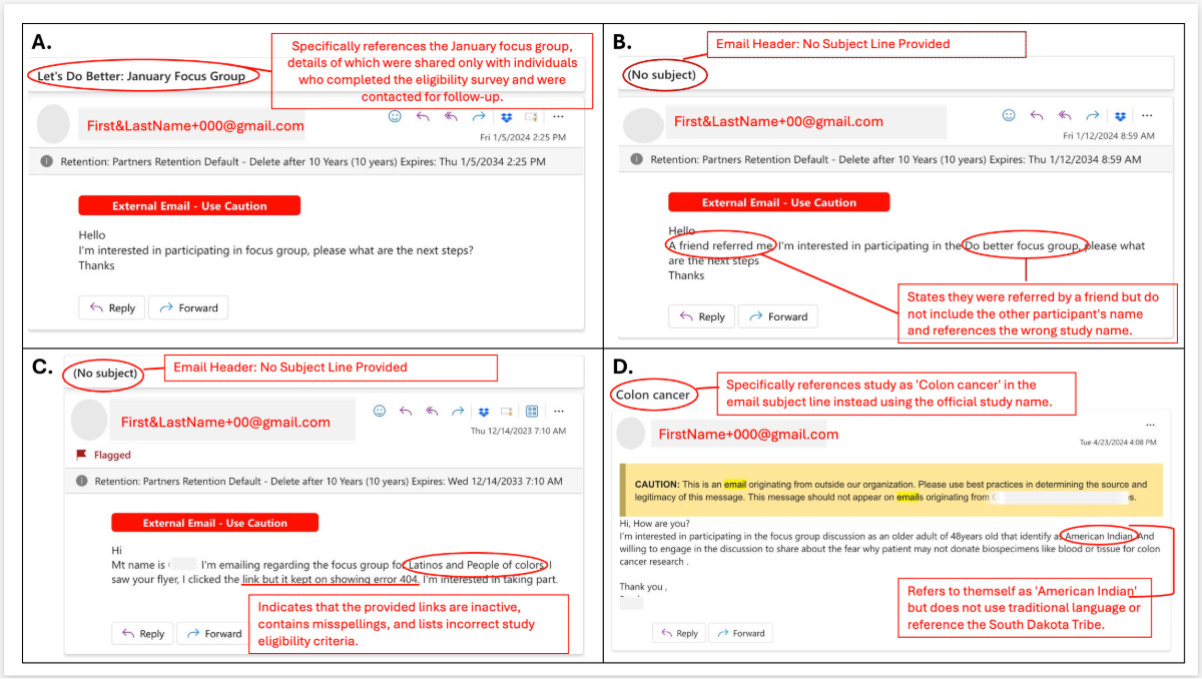
Examples of suspicious email communications received from potentially fraudulent participants encountered during study recruitment in Boston, MA, and South Dakota, United States, 2024. (A) Email from uninvited participant requesting to join the nonadvertised focus group session. (B) Email from an unverified participant claiming they were referred to the study but using the wrong study name. (C) A potentially fraudulent participant claims they saw the study flyer online, but with error messages preventing confirmation of eligibility. (D) Email received at the South Dakota site by staff, but it did not use the traditional language used among the tribal population.

In South Dakota, fraudulent participants were identified by several distinct patterns in communication and behavior by the research study team. Emails from suspected fraudulent participants frequently contained phrasing and language inconsistent with the local community’s unique communication style (Table 1). These emails often had generic (eg, “colon study” or “colon cancer”) or off-topic subject lines (eg, “suicide prevention”) and included statements like, “I identify as American Indian” (Figure 1D). However, as stated by an enrolled tribal member of the Great Plains Tribal Health staff, it is customary to proudly identify by tribal affiliation rather than using generic terminology. The email messages we received displayed clear indications of being copy-and-pasted, with identical text across multiple individuals (Figures 1A-1D).

In Study 2, investigators noted that more than half of the participants interested in the study individuals completed the consent process within the same 3-hour window. Beyond demographic inconsistencies, those who enrolled during this period overwhelmingly reported unusual personal and family health histories inconsistent with prior enrollments. For example, the investigators found that more than half of these participants indicated they were previously diagnosed with cancer in their survey. Similarly, multiple focus group participants who completed the sessions provided responses that were inconsistent with their answers to the initial eligibility survey. In addition, other participants obscured their faces, turned off their cameras and audio, and were only willing to participate via the Zoom chat. Only when focus group leaders advised that participation by turning on cameras and/or audio was necessary for remuneration, were participants then hesitantly willing to turn on their cameras and/or microphones, often partially or entirely obscuring their faces, raising further suspicion of fraudulent participation.

### Remediation Strategies

To address these issues and ensure data integrity, Study 1’s Boston site implemented several corrective measures. Even if participants appeared eligible based on the survey screener, their contact information was scrutinized for patterns matching those of known fraudulent participants (Table 3). This included checking for the use of Google Voice numbers by using a phone carrier lookup tool that indicates when a number is via Google Voice services. The study team member also checked for unusually fast survey completion times and email address formatting that aligned with previous suspected fraudulent cases. Study 1’s Los Angeles site implemented several remediation strategies to enhance participant verification in response to their challenges. A secondary point of contact, via email or phone communication, was introduced as an additional screening step between initial expressions of interest and official focus group enrollment. The study team also ceased posting recruitment links on social media platforms to reduce exposure to potential fraudulent participants. Finally, the consent form was revised to explicitly state that participants would be required to have their cameras on during focus group sessions as a measure to ensure authenticity and engagement.

To verify authenticity of participants in Study 1’s South Dakota site, follow-up replies were sent, requesting that individuals call directly to complete enrollment. Calls from suspected fraudulent participants often originated from out-of-state area codes, and callers frequently spoke using phrasing or words that suggested they were not from the local community. They would often ask generic questions such as, “I’m interested in participating in the focus group discussion as an older adult of 48 years old that identifies as American Indian,” a sharp contrast to the community members’ more personalized inquiries (Figure 1D). Other participants, in contrast, engaged directly via phone, completed demographic and eligibility questions, and provided the necessary information to be entered into the REDCap database. This distinction in communication style and procedural follow-through made the team identify them as potentially fraudulent and proceed to filter them out of the enrollment process.

Study 2 investigators were able to discern potentially fraudulent participants from eligible ones by implementing several strategies. First, individuals who appeared to have completed the eligibility questionnaire multiple times in quick succession were identified for closer investigation. The dramatic periods of increased enrollment rates, which did not correspond with any increase in outreach activities, were scrutinized to pinpoint and exclude fraudulent participants (Table 1). Next, investigators reviewed data to ensure consistency in responses, assessing both survey answers and focus group contributions for coherence and reliability. For example, participants that indicated on the eligibility questionnaire that they had undergone genetic testing, but then indicated in focus groups they had not, were noted for further review. Researchers also confirmed that the addresses provided were associated with residential locations rather than businesses and examined participants’ emails that were formatted in similar styles, which helped in identifying legitimate participants.

**Table 3.** Prevention, detection, and remediation strategies for fraudulent and imposter participants.

Prevention or remediation strategies	Purpose	Fraud type
Restrict access to meeting links	Do not publicly post web conference links. Provide personalized meeting links to verified participants whenever possible to prevent unwanted guests in focus groups or interviews.	Duplicate enrollee, ineligible or unverified enrollee, bot, and meeting crashers
Waiting room/closed rooms	A waiting room is a virtual staging area where participants remain before being admitted into a Zoom (Zoom Communications, Inc) meeting. A closed room restricts access to only preapproved participants.	Duplicate enrollee, ineligible or unverified enrollee, bot, and meeting crashers
Contact information verification	Require verifiable phone numbers (Google Voice; calls originating from the targeted region of study) or emails to confirm participant authenticity.	Duplicate enrollee, ineligible or unverified enrollee, bot, and meeting crashers
Review survey completion time	Monitor completion times to flag respondents who answer suspiciously fast. For example, you could set a minimum threshold for expected survey duration and flag responses completed too quickly for further review.	Duplicate enrollee, ineligible or unverified enrollee, bot, and meeting crashers
Require cameras on during focus groups or interviews	Include in enrollment process that participants will need to be in an isolated location with cameras on throughout the entire session	Duplicate enrollee, ineligible or unverified enrollee, bot, and meeting crashers
Clarify and confirm	Duplicate enrollments may not be intentional acts of fraud. It may result from confusion or misunderstanding. State that each person can only enroll once in recruitment and consent documents.	Duplicate enrollee, ineligible or unverified enrollee
Cross-check validity	Cross-check new interested individuals with those already screened or enrolled before proceeding.	Duplicate enrollee, ineligible or unverified enrollee
Enable survey antifraud reCAPTCHA^a^.	reCAPTCHA tools present tasks like image identification, simple math equations, or deciphering distorted text. These tasks are designed to be easy for humans, but difficult for bots. Enable these tools for all public surveys and screeners.	Bot
Attention checks	Embed verification questions to assess attentiveness and detect random or automated responses. Example: “Please select ‘Strongly Agree’ for this question”	Bot
Reverse-coded and unexpected questions	Use questions that contradict expected responses or randomize question order per participant to prevent automated scripts from predicting patterns. For example, “Have you won the lottery recently?”	Bot
Limit public advertising of incentivized studies	Consider public-facing recruitment materials on social media platforms carefully, including potentially not listing financial incentives.	Bot

a CAPTCHA: completely automated public turing test to tell computers and humans apart.

### Impact

Across all sites, the implementation of remediation strategies to minimize suspected imposters or fraudulent participants led to notable changes in the recruitment process and focus group sessions. At the Boston site in Study 1, once a potentially fraudulent participant was identified due to having a Google Voice number, they were flagged in internal/external recruitment platforms and in REDCap as potential fraud. These suspected imposters did not receive a call back to engage with the study any further. At the Los Angeles site, the removal of social media recruitment links combined with additional phone call verifications resulted in fewer responses and a reduced number of fraudulent survey submissions and potentially fraudulent individuals in focus group sessions. In Study 2, the study team took the decision to conduct real-time analysis after observing a dramatic increase in enrollment. This allowed them to identify who was either a fraudulent or imposter participant within the focus group sessions. Subsequently, those individuals were removed and/or the focus group session was terminated early due to failure to adhere to the eligibility requirements to be in the space.

### Oversight and Reporting

#### Recruitment Platforms

In response to the rising concerns about fraudulent participant activity, Study 1’s Boston site reported the suspicious users that attempted to enroll to their internal recruitment platform (Table 4). Approximately 2 months after the end of focus groups at the Boston site, the platform sent a notice titled “Help us Fight Spam.” This notice acknowledged an ongoing issue they were having with suspicious users on the platform, which led to the development and implementation of a “spam” indicator that study administrators could use to flag suspicious or problematic accounts on the platform. This new detection tool allowed Study 1’s Boston site to go back through their records to mark imposter or fraudulent individuals as “spam” and leave a note explaining to report that account and prevent them from engaging in further studies.

**Table 4.** Oversight and reporting: recommendations for action after infiltration of imposters in your study.

Entity or institution	Recommended actions	Anticipated results
Institutional review board	Include plans for preventing, identifying, and removing imposters in your IRB^a^ protocol. Report protocol violations to IRB. Enrolling ineligible individuals or more participants than expected (due to bots) may be protocol violations. Check with your IRB and report as mandated	IRBs may request changes to recruitment or screening procedures by including a standard operating procedure structured for recruitment, data collection, and consent process.
Funding sources	Inform funders through regular reporting processes (eg, progress or semiannual reports) when imposter or fraudulent activity is suspected. Provide them with steps taken to mitigate fraudulent activity and exclusion of affected data if applicable.	Funding agencies may support remediation strategies that will maintain the study integrity and account for transparency.
Study recruitment platforms	Report potentially fraudulent users to the study recruitment platforms in real time. Provide them with all necessary details of how they were fraudulent so they can assess their profile and others that may be deemed similar.	Platforms may implement new screening tools or alerts for researchers to detect fraudulent submissions
Advertisers (eg, social media, …)	Contact the platforms' support team directly to report fraudulent activity from suspected accounts.	Platforms may report those accounts and enhance security measures for those users.
Police and legal authorities	Assess if the suspected fraudulent activity requires police or institutional legal counsel. Document the decision not to involve police or legal counsel and continue with internal oversight or IRB guidance.	Allows for appropriate escalation pathways to be considered, while also continuing to use internal oversight as the primary reporting mechanism.
Presentations and manuscripts	Include imposters in flow diagrams and charts. Describe approaches implemented to prevent, identify, and remove imposters from your study	Allows the research community to see actual examples of phone conversations and email responses that can be used as an indicator or language to look out for. Resources and tools to help people understand.

a IRB: institutional review board.

#### IRB

Study Team 2 reported the suspected fraudulent activity to their local IRB in accordance with their protocol (Table 4). After careful review, the IRB determined that since thematic saturation had already been reached by the time fraudulent activity was suspected, the affected data would be removed from the overall analysis to preserve the integrity of the study. The IRB also advised that all participants who had completed the one-on-one cognitive interviews or focus group procedures should be remunerated, regardless of whether their participation was later identified as fraudulent, as there were no provisions for withholding remuneration in their study protocol. Following these informed discussions, Study Team 2 amended their protocol to include the above-mentioned approaches to prevent future instances of fraudulent participation in subsequent parts of the study.

#### Funding Sources

In Study 1, during the project’s semiannual reporting process, the funder was informed that this manuscript was being developed as a subset of the initial project. Data contributed by individuals who were suspected to be fraudulent were excluded from the final analysis.

As previously described, in Table 1, investigators from Study 2 reported suspected fraudulent activity to their IRB, which outlined how the study could proceed given the potentially fraudulent or imposter participants. The study funder was informed of the IRB determination and supported the approach that was recommended.

#### Police and Legal

Across both studies, police and legal authorities were not notified of the suspected fraudulent or imposter participants, as no laws were essentially violated.

## Discussion

### Principal Findings

As seen in the cases discussed, the impact of such deception was not isolated incidents but rather showcased systemic vulnerabilities in how we conduct outreach and enrollment in virtual qualitative research studies [6]. Across 2 multisite studies, we identified recurring evidence of fraud, including highly repetitive email addresses, unusual email and phone responses, and atypical language patterns. One main vulnerability was advertisement (including information about incentives) via public-facing websites and use of research platforms that may not require account verification as well as social media platforms [12]. While the inclusion of these methods of recruitment aimed to broaden accessibility, they inadvertently increased our exposure to fraudulent or imposter individuals. Balancing the use of digital methods for recruitment with security requires rigorous validation that can pose a challenge to participant privacy and inclusivity. We know that more restrictions and eligibility criteria tend to decrease access and equity [6,7,23]. We must ensure that fraud detection measures do not disproportionately exclude genuine participants, particularly those from underrepresented or marginalized groups [24].

Our experiences aligned with Medero et al [12], who also reported infiltration of fraudulent participants in qualitative research. While their study focused on 2 qualitative case studies, one involving Black youth and mental health and the other examining Black birthing individuals’ experiences, our research spanned multiple sites and designs. Despite these differences, both studies encountered similar indicators of fraud, patterned email addresses, unverifiable Google Voice numbers, rushed eligibility responses, and unusual behavior and language during virtual focus groups. A key takeaway is the critical need for researchers to adopt a multifaceted approach to fraud prevention and recognition during study design and implementation. While technological solutions like completely automated public turing test to tell computers and humans apart (CAPTCHA) challenges and digital fingerprinting are valuable, they are not sufficient on their own [25]. A combination of presurvey checks, cross-validation methods, and ongoing collaboration with online platforms and institutions is essential to maintaining data integrity [26,27].

### Recommendations for Future Studies

We offer the following recommended strategies for how researchers may prevent, detect, and address fraudulent and imposter participation in their studies in the following: (1) incorporate fraud detection methods at baseline; (2) conduct presurvey and postsurvey validity checks; (3) report fraudulent or imposter enrollment in studies to the IRB; (4) collaborate with social media platforms to report fraudulent accounts and bot activity; and (5) share your experiences with imposters and fraudulent participants when you report study results. We describe each recommendation in greater detail in the subsequent paragraphs.

Using advanced screening techniques, such as CAPTCHAs, reCAPTCHA’s, and 2-step authentication, may significantly reduce fraudulent users’ completion of eligibility surveys [28]. As shown by Storozuk et al [26], these measures are not infallible due to CAPTCHA codes being easily diverted, but they can be used as a screening technique to identify a specific type of fraudulent behavior that uses bots’ software programming. Second, researchers should develop and include in their research protocols a plan for real-time analysis of recruitment data during data collection to help identify fraudulent participants [23]. This plan should include monitoring for excessively rapid survey or screener completion times, a high volume of responses submitted over an unusually short period, and inconsistent survey responses or unusual response patterns, all of which may signal deception or fraudulent activity. Third, investigators and study teams need to report imposter or fraudulent occurrences to their IRB. By reporting incidents of fraudulent participants to the IRB, researchers can be better informed on how to complete their data collection while also increasing the IRB’s knowledge of intervention methods to include in the initial review of study applications [24].

Increasing partnerships with online research survey platforms is essential to enforce new system protocols for users creating accounts to interact with research studies. These partnerships, as highlighted by Dewitt et al [27], can lead to the implementation of robust validation mechanisms, including recontact procedures, to verify participant identities and improve response accuracy. Working in tandem with these software platforms and reporting users who may be fraudulent to their administrative support teams can indicate measures of where their systems are vulnerable to fraudulent users. Finally, it’s crucial that researchers and institutions are transparent about and thoroughly document their research process. Study teams should be able to recount what happened, why it happened, and the decisions made to better inform fraud detection practices that can be used in future studies. This information can and should be included in publications reporting the results of such studies. This allows other researchers to learn from their experience and increases overall awareness across the research community on how to detect fraudulent users to address the attacks on data manipulation in clinical research settings.

### Limitations

Several limitations should be considered. In the studies described here, most determinations about whether a participant was potentially fraudulent relied on circumstantial evidence and the research team’s judgment rather than factual evidence, leaving room for error. While we used a variety of platforms developed to have detection tools that ranged from CAPTCHA, Zoom safeguards, and verification questions, we could not be certain that every participant that we flagged as “fraudulent” was indeed so. It is also possible that some fraudulent or imposter participants were able to evade detection. These limitations highlight the broader challenge of identifying clear thresholds that determine when a participant is considered fraudulent or an imposter. Future researchers must be vigilant of this risk and adopt a proactive approach to identify and prevent fraudulent activity during recruitment and enrollment. As such, this is particularly important in qualitative and mixed methods studies, where each participant’s contribution can significantly influence the study’s findings.

### Conclusion

Our multisite analysis demonstrates that fraudulent and imposter participants pose a significant and evolving threat to the integrity of qualitative and mixed methods research. As web-based recruitment and data collection become more prevalent, researchers should take additional steps to identify fraudulent actors and reduce their influence. Our experiences across 2 studies and multiple sites reveal that deception can manifest in various forms, from bots and duplicate enrollees to ineligible individuals. These types of fraudulent actors are capable of distorting data and undermining research credibility. Researchers need multiple verification measures, real-time data analysis, and a comprehensive approach that collaborates with recruitment platforms, the IRB, and the research community to effectively address this issue. By implementing robust preventive strategies and fostering collaboration among researchers, we can begin to mitigate these occurrences and implement safeguards that increase the integrity of our research.

## Supplementary material

10.2196/87037 Multimedia Appendix 1 Examples of flyers and social media advertisements used across both studies were potentially fraudulent and imposter activity took place.

## Abbreviations:

CAPTCHA: completely automated public turing test to tell computers and humans apart
IRB: Institutional Review Board
REDCap: Research Electronic Data Capture
