# Supplementary material for: Detecting and Preventing Fraudulent Participation in Qualitative Research: Content Analysis of Two Multisite Studies
Source: J Med Internet Res. 2026 Jul 3;28:e87037. doi: 10.2196/87037 (PMC13331394; doi:10.2196/87037)
Supplement: Multimedia Appendix 1 [file jmir-v28-e87037-s001.pdf]

Multimedia Appendix: Online Resources 1- 7

Detecting and Preventing Fraudulent Participation in Qualitative Research: Content Analysis of Two Multisite Studies

Journal of Medical Internet Research

Corresponding Author

Erica T. Warner

100 Cambridge Street

Boston, MA 02114

Tel 617-724-9516

Email: ewarner@mgh.harvard.edu

Online Resource 1 – SU2C MGH Boston Site English Flyer (Study 1)

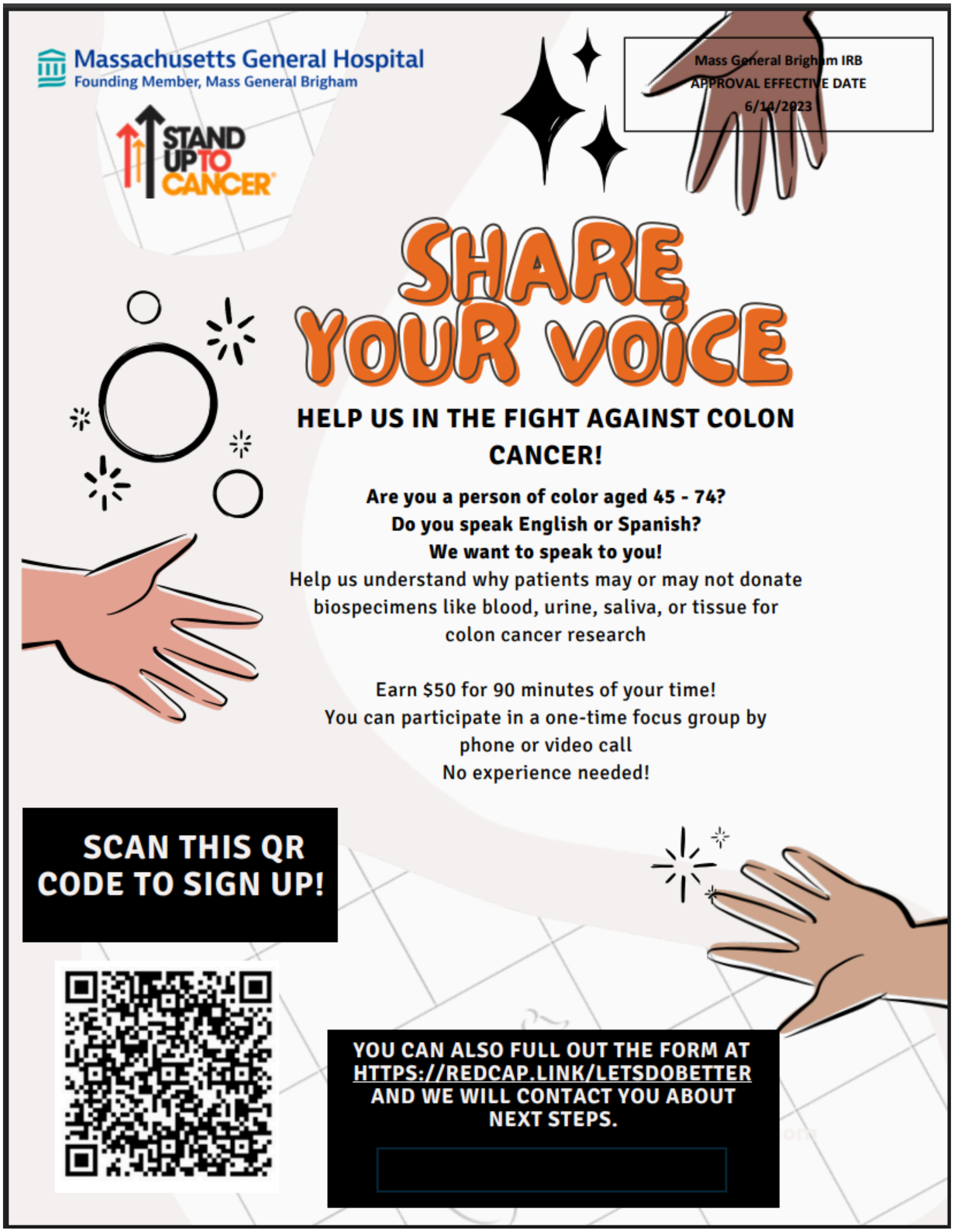

Online Resource 2 – SU2C MGH Boston Site Spanish Flyer (Study 1)

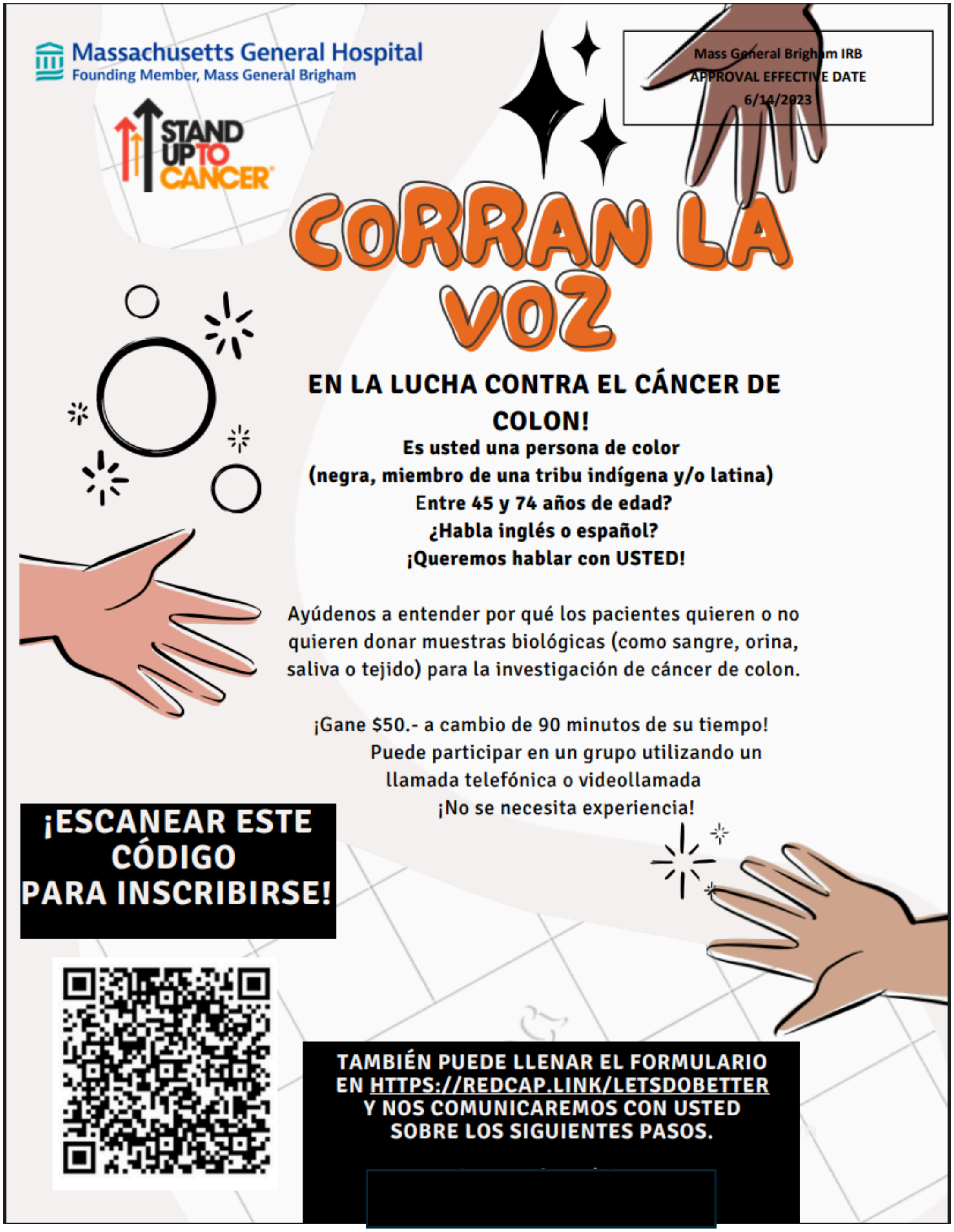

Online Resource 3 - Stand Up to Cancer Research Match Message Advertisement Boston Site (Study 1)

You are invited to participate in a research study titled *Let's Do Better: Community and Patient Centered Approaches to Biospecimen Research*. Through a one-time focus group, we would like to learn more on how to include more people from Black, Hispanic or Latino/a/x, and Native American communities to be involved in future research to donate samples like saliva, blood, stool or tissue. This will help us improve our ability to prevent or detect earlier forms of diseases like cancer, which disproportionately harm communities of color.

By participating, you could help us learn how we can design effective strategies to enroll Black, Hispanic or Latino/a/x, and Native American communities in research where we plan to collect blood and stool to study colon cancer prevention.

You may qualify for this study if you:

- Are age 45-74
- Identify as Black, Hispanic or Latino/a/x, or Native American
- Speak English or Spanish
- Have never been diagnosed with colon cancer

This study involves:

- A one-time online focus group that will last between 60-90 minutes.
- Each participant will receive a $50 gift card.

Online Resource 4 – Internal Institutional Research Website Advertisement Boston Site (Study 1)

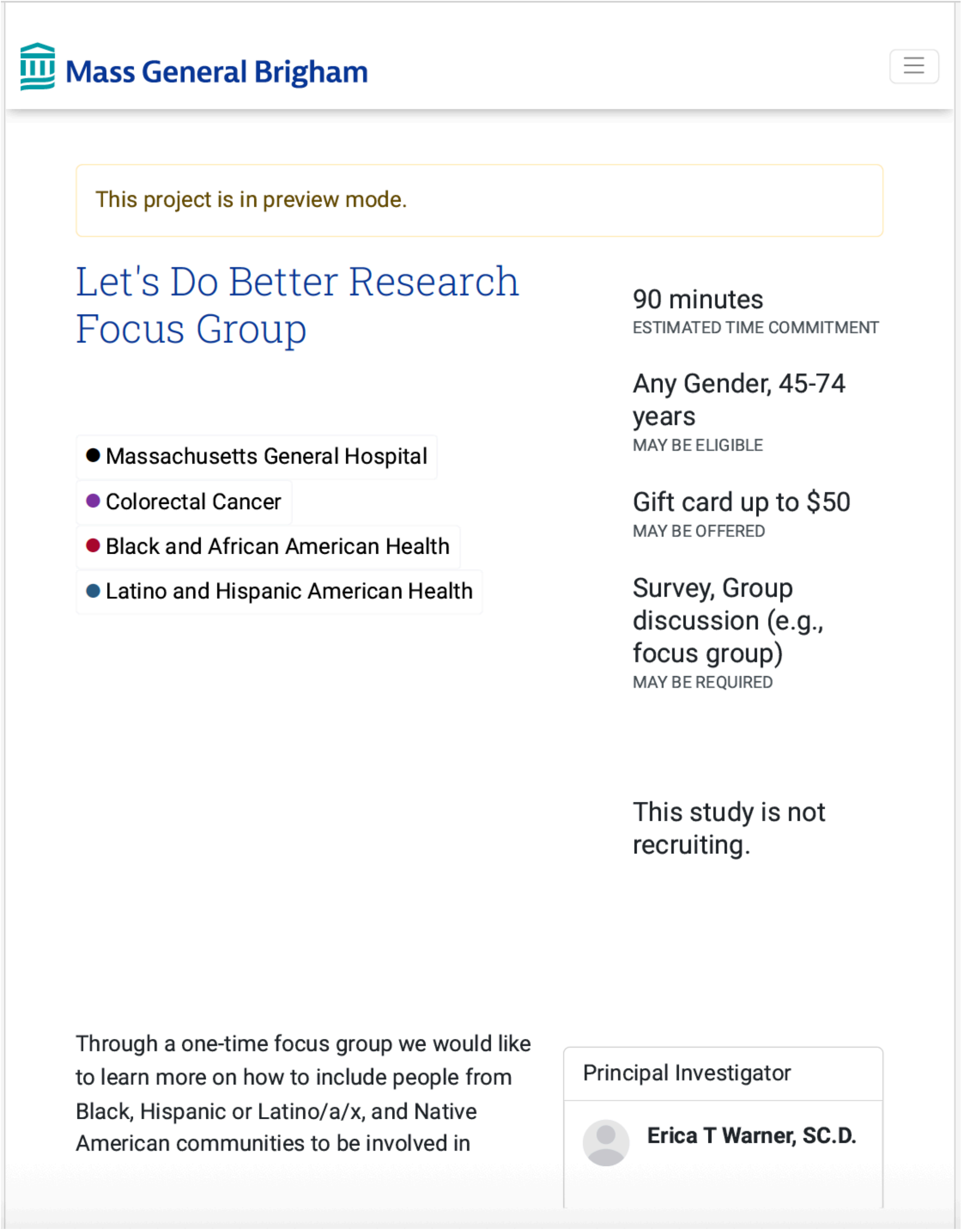

future research to donate samples like saliva, blood, stool or tissue.

Massachusetts General Hospital

Public profile

What we are studying

We need more people from Black, Hispanic or Latino/a/x, and Native American communities to be included in research to donate samples like saliva, blood, stool, or tissue. This will help us improve our ability to prevent or detect earlier forms of diseases like cancer, which disproportionately harm communities of color.

What we hope to accomplish

We are interested in learning from you how we can design effective strategies to enroll Black, Hispanic, or Latino/a/x, and Native American in research where we plan to collect blood and stool to study colorectal cancer prevention.

Who can participate

Participants who: Age 45-74, Identify as Black, Hispanic, or Latino/a/x, or Native American/American Indian, and have no personal history of colon cancer.

☑ Healthy Volunteers

Healthy volunteers are eligible for this study

Who cannot participate

Participants who: Have a personal history of colon cancer. Adults unable to consent and prisoners will be excluded. Infants, children, and teenagers do not meet age eligibility criteria for this study and are not included.

What participants may be asked to do

You will be asked to fill out a brief survey and attend a one-time virtual focus group held via Zoom.

Project activities may include:

- Survey
- Group discussion (e.g., focus group)

Estimated time commitment

90 minutes

What participants may get

You would recieve a $50 gift card via mail or email after completion of the focus group.

Online Resource 5 – SU2C Los Angeles Site English Flyer (Study 1)

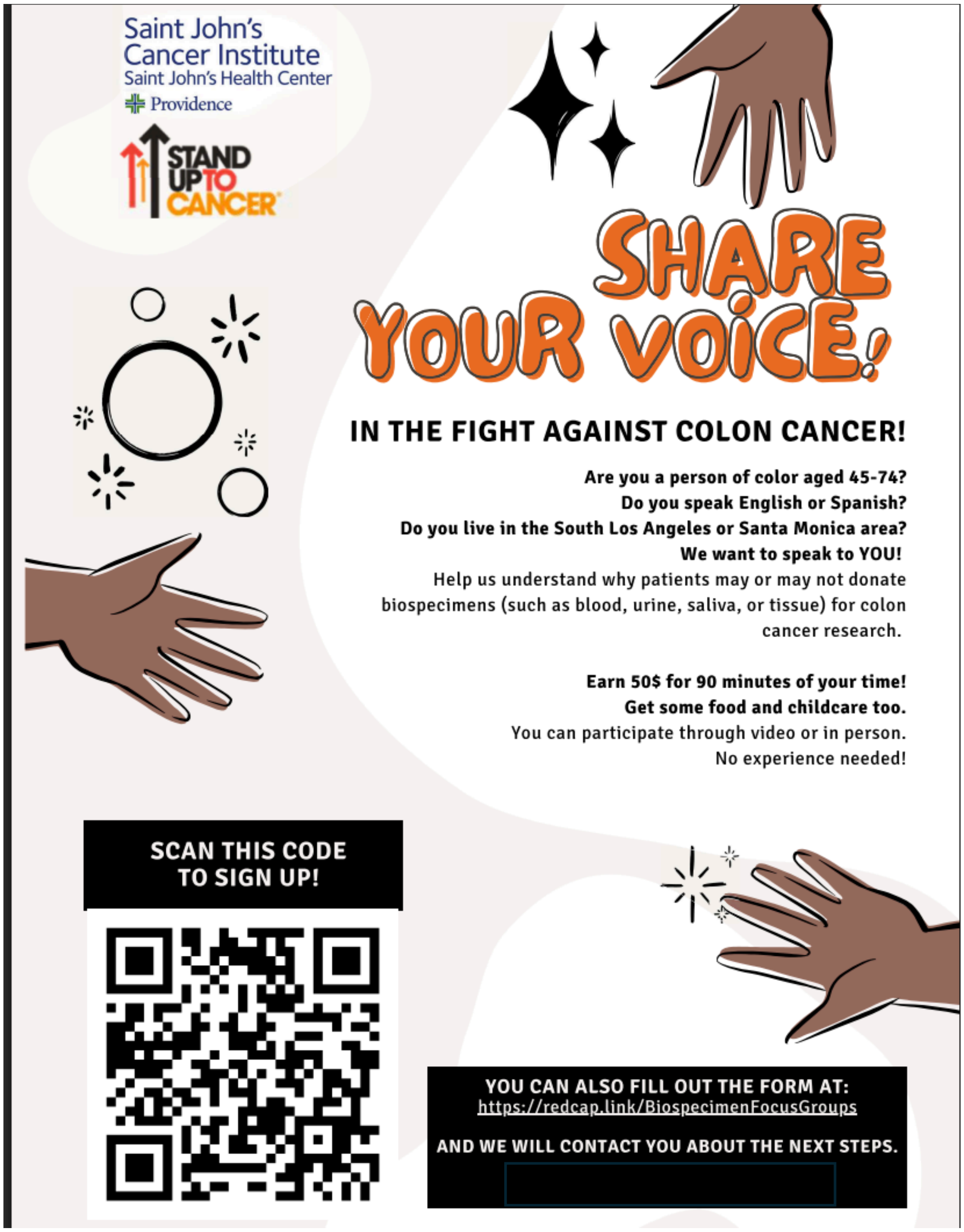

Online Resource 6 – SU2C Los Angeles Site Spanish Flyer (Study 1)

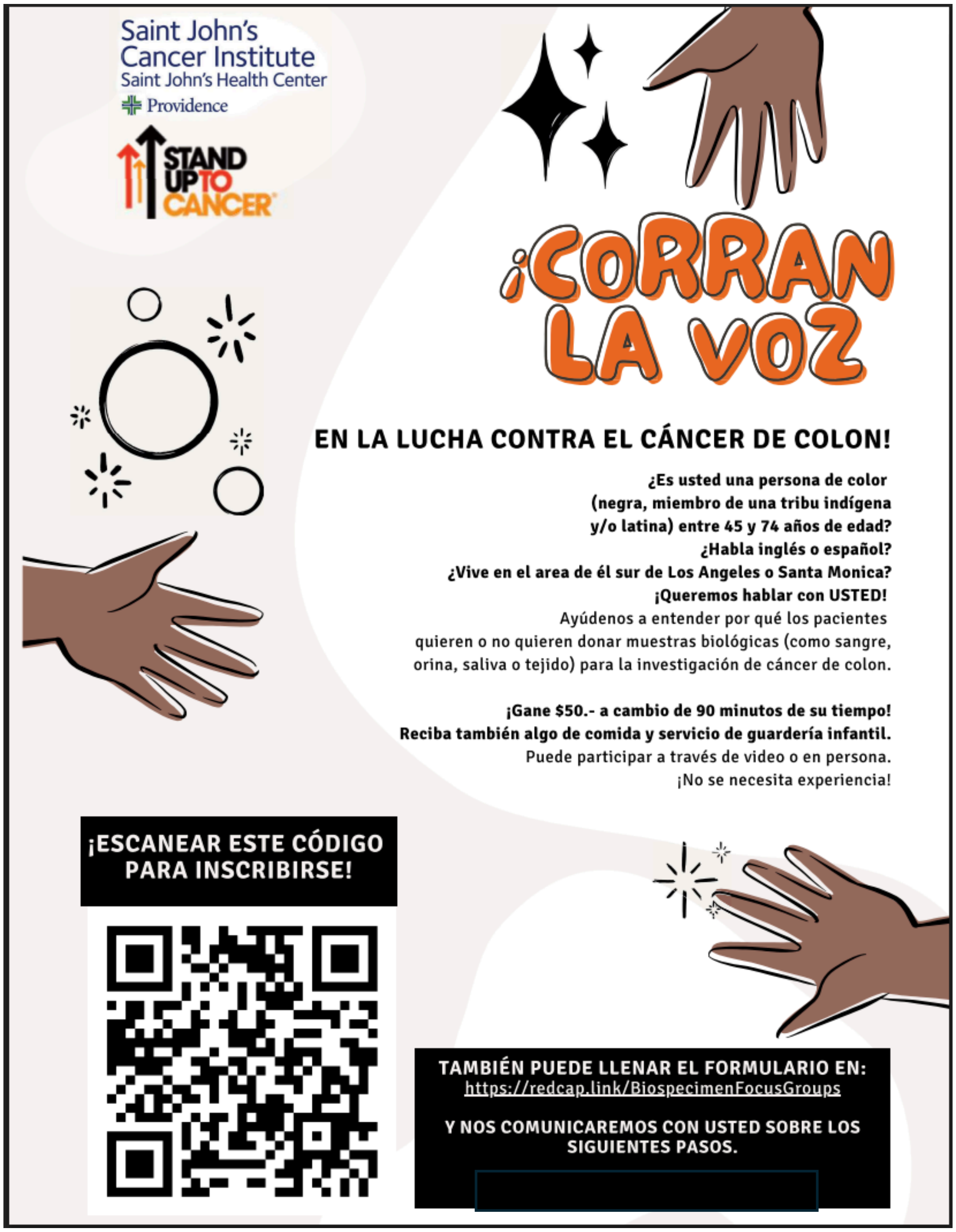

Online Resource 7 – SU2C South Dakota Site Flyer (Study 1)

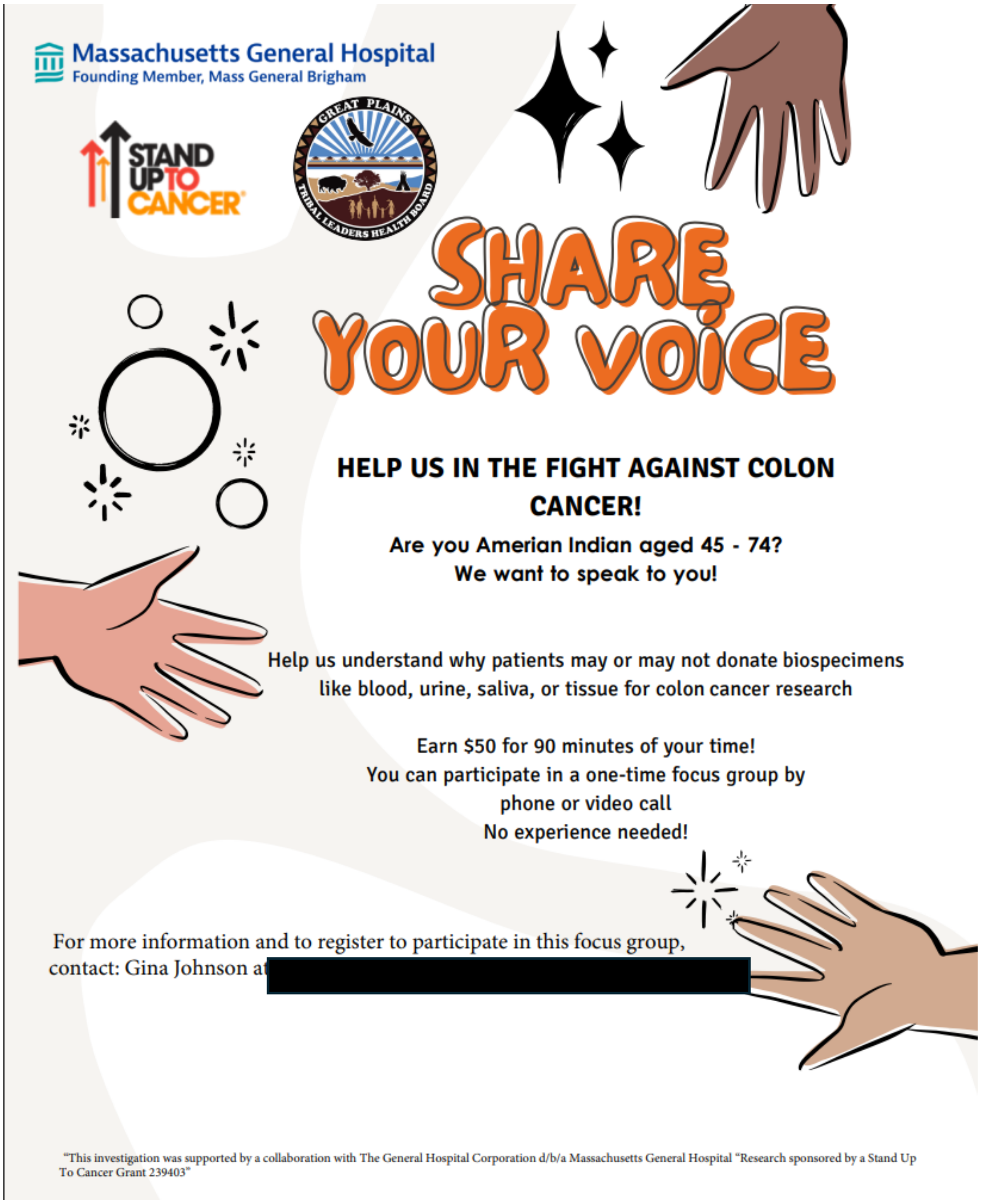

Online Resource 8 - Social Media Content (Study 2)

Heading: Adult Research Volunteers Needed for Focus Group Study

Content: We are looking for Black and Latin/Latino/Latina/Latinx volunteers as well as community partners (patient advocates, community leaders, healthcare providers, and community health workers.) that serve these communities, to share their ideas about how to improve our approach to genetic education, genetic testing, and cancer screening through focus group discussions. A focus group is a guided conversation, involving a small group of people.

Genetic testing can help prevent disease in some cases. We are trying to learn about different ideas about getting genetic education, genetic testing, and cancer screening. We want to use the information we learn to create an online genetic education system that will be used members of the community to access genetic education and testing more easily.

No genetic counseling or genetic testing is involved. No computer skills are required but access to a computer, phone or tablet with internet is needed. You must be available for a one-time online focus group discussion that will last 60-90 minutes. Participants will receive a $50 gift card upon completion of the task. If you speak English or Spanish and are interested, please contact us to see if you are a good fit for this research study. You may also be invited to participate in a follow up one- on-one interview.

<Study contact information>
